# Design of a dynamics-based hydraulic controller for lifting manipulator wrist and its stability analysis

**DOI:** 10.1371/journal.pone.0347838

**Published:** 2026-04-28

**Authors:** Bobo Li

**Affiliations:** Furong Craftsman College, Hunan Industry Polytechnic, Changsha, China; King Fahd University of Petroleum & Minerals, SAUDI ARABIA

## Abstract

Under the requirements of Industry 4.0, the performance requirements for improving the hydraulic controller of the mechanical hand wrist are getting higher and higher. To address issues such as response delay and insufficient accuracy in traditional methods, this study proposes a hydraulic control based on a dynamic model. The core innovation lies in embedding the dynamic model into the control loop and integrating multiple intelligent algorithms for closed-loop optimization. Experimental verification shows that the maximum trajectory tracking error of the SHD controller is only 0.28 mm, the fault detection accuracy is as high as 98.3%, and the energy conversion efficiency is 98.1%. It is significantly superior to existing advanced controllers in terms of accuracy, stability, and response speed, such as the controller combining quantum-inspired neural networks with robust control, the controller combining meta-learning with fuzzy wavelet control, and the controller combining federated learning with edge control. The above research results provide an efficient solution for the precise and intelligent control of hydraulic systems in complex lifting operations.

## 1. Introduction

The key to accurate and safe operation is the hydraulic control technology of a lifting robot arm’s wrist. It is crucial in critical situations like handling large objects, precise assembly, and operating in dangerous areas [1]. Its performance directly determines the robotic arm’s operational efficiency, accuracy, and overall safety [2]. Therefore, many scholars have conducted in-depth analyses of this. Z. Wang et al. proposed a pressure compensation disturbance suppression control strategy based on an extended state observer to improve the performance of the secondary regulation system under variable pressure conditions. The experimental results show that under the condition of system pressure fluctuations, the proposed method improves control accuracy by 55.2% and 32.1%, respectively, compared to traditional PID control and adaptive robust control methods [3]. X. Ren et al. proposed a novel specified performance fault-tolerant control method for electro-hydraulic servo systems with actuator faults, external disturbances, and unknown nonlinearity. The effectiveness of the proposed method was verified through simulation and experiments conducted on the electro-hydraulic servo system platform [4]. Junhui Zhang et al. developed an identification framework to address the problem of insufficient control performance of hydraulic controllers. The framework effectively improved the control performance of hydraulic controllers by integrating the physical constraints imposed by inertia, friction, and hydraulic parameters. The proposed framework reduced the tracking error rate by 50%–56% [5]. Y. Sun et al. developed an impulse interference compensation controller based on the backstepping method. A large number of comparative experiment results show that after adding SDCC, the maximum tracking error and variance of PID were reduced by an average of 68.7% and 68.55%, respectively [6]. However, the performance ceiling of the above-mentioned framework is limited by the model recognition accuracy, and its adaptability and robustness face challenges when dealing with unmodeled dynamics and sudden failures. J. Ye et al. designed a switching dynamic framework to simulate the exchange phase and developed a comprehensive position control scheme for heavy vessels. The effectiveness of the proposed scheme was verified on a realistic heavy ship simulation platform, and the results showed that compared with the non switching scheme designed for only one stage of the construction scene, the switching framework proposed significantly improved accuracy and reduced danger [7]. J. Ye et al. proposed a new DP method consisting of an observer and a controller, and tested the proposed method in an integrated crane simulation environment, where the integration of several subsystems (winch dynamics, crane force, thruster dynamics, fuel injection system, etc.) allowed for real-world validation under a wide range of uncertainties. The results show that this method has stability assurance in the presence of uncertainty [8]. Most of the existing studies lack the collaborative design of advanced algorithms based on dynamic principles and hardware systems. They often solve problems in isolation and find it difficult to address the interwoven challenges, such as accuracy, stability, adaptability, and intelligent fault diagnosis of hoisting robotic arms under complex dynamics as a whole. In contrast, this study has achieved closed-loop dynamic optimization from trajectory planning to actuators through a multi-algorithm collaborative architecture. It not only overcomes the response delay problem of traditional methods but also surpasses the limitations of a single intelligent strategy in overall control performance.

The lifting system exhibits strong coupling and uncertainty due to multi-joint coordination and dynamic load variations, imposing stringent requirements on the real-time performance and accuracy of the hydraulic controller. Traditional methods struggle with extreme loads and nonlinear dynamics, leading to issues like slow response and control deviations that compromise efficiency and safety. To address these limitations, this study proposes a novel hydraulic control model based on dynamic principles by integrating multiple intelligent algorithms. Specifically, the Unscented Kalman Filter (UKF) provides precise state estimation, the Adaptive Neuro-Fuzzy Inference System (ANFIS) handles uncertainties and nonlinearities, the Convolutional Neural Network (CNN) extracts fault features, and Model Reference Adaptive Control (MRAC) compensates for disturbances in real-time. This integrated approach ensures enhanced stability, adaptability, and overall control performance.

The innovation of this study lies in: (1) deeply embedding the dynamic model into the control loop to achieve closed-loop optimization from trajectory planning to execution; (2) Designed a multi algorithm collaborative control architecture that integrates UKF, ANFIS, CNN, and MRAC; (3) Using a central control logic unit to coordinate data sharing and collaborative decision-making among all modules.

The contribution of this study lies in: (1) improving the adaptability of the controller to complex dynamic environments; (2) Integrated design has been achieved, enabling real-time decision-making based on actual system dynamics and achieving closed-loop dynamic optimization from perception to execution; (3) We have developed a complete intelligent control system that can effectively address dynamic challenges under complex working conditions, significantly improving trajectory tracking accuracy, fault diagnosis capabilities, and system stability. The purpose of the research is to solve the problems of response delay and insufficient accuracy of existing hydraulic control methods under complex working conditions, and to improve the reliability and safety of hydraulic systems.

## 2. Methods and materials

### 2.1. Design of dynamics-based hydraulic controller for lifting manipulator wrist

**Fig 1:**
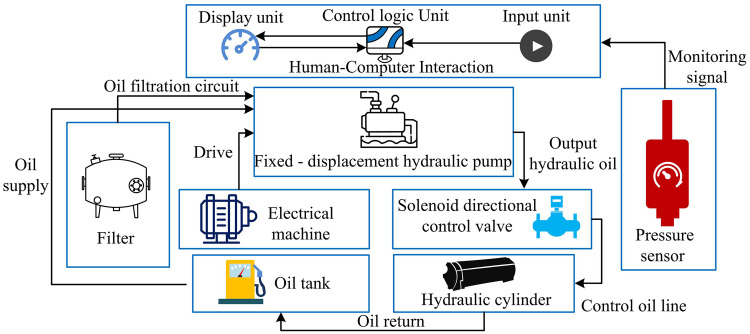
Dynamics-oriented hardware system architecture (Icon source from: https://icon.sucai999.com/).

The hydraulic controller for the lifting manipulator wrist is a dynamics-based core unit, powered hydraulically and integrating sensors and algorithms for precision lifting of heavy loads. Dynamics-guided hardware design ensures accurate matching between hydraulic components and the manipulator’s mechanical requirements. Traditional systems with fixed-displacement pumps and cylinders, while structurally simple, lack adaptability and suffer from poor real-time performance due to manual parameter tuning [9–10]. To design a model-based controller, a dynamic model of the wrist of a lifting robotic arm was first established. This model comprehensively considers joint angle, angular velocity, angular acceleration, and dynamic load changes, and uses the Lagrange method to construct system dynamics equations, providing a theoretical basis for controller design. On this basis, the SHD hydraulic controller architecture was proposed. Therefore, the study embeds the dynamics-oriented module into the hardware system, as shown in the structural diagram in Fig 1.

In Fig 1, this dynamics-based hardware system uses a motor to drive a fixed-displacement hydraulic pump, which draws oil from the tank, purifies it through a filter, and outputs hydraulic oil. A solenoid directional valve, controlled by a dynamic control logic unit, adjusts the oil flow to drive the hydraulic cylinder and actuate the manipulator wrist. A pressure sensor collects real-time oil pressure data and sends dynamic feedback signals to the human–machine interaction module. Operators send commands via the display and input units. The control logic unit processes these commands to dynamically adjust components such as the solenoid directional valve. After completing the cylinder movement, the oil returns to the tank, forming a cycle. The system applies dynamic algorithms to optimize each process, achieving stable and precise control of the manipulator wrist. The output force of the hydraulic cylinder is calculated as shown in Equation (1) [11].


$$F=A\times (p-p_{b})-F_{f}-m\times a$$ (1)

In Equation (1), F represents the output force of the hydraulic cylinder, unit: N. A is the effective area of the cylinder piston, unit: m^2^. p is the pressure in the working chamber, unit: Pa. $p_{b}$ is the back pressure, unit: Pa. $F_{f}$ represents the friction during cylinder motion, unit: N. m and a denote the mass and acceleration of the moving components, with units of kg and m/s^2^ respectively. The output voltage of the pressure sensor is calculated as shown in Equation (2).


$$U=U_{\text{0}}+k\times p\times \left(1+k_{T}\times \left(T-T_{\text{0}}\right)\right)$$ (2)

**Fig 2:**
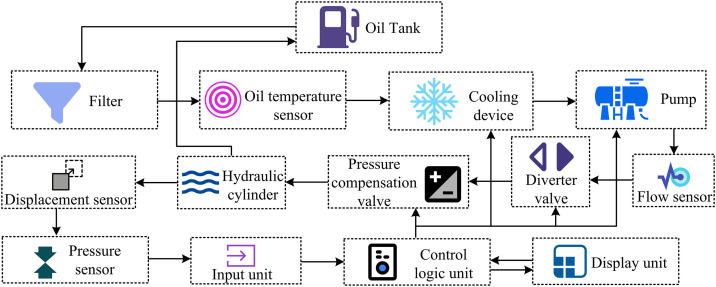
Operational flow of improved hardware system (Icon source from: https://icon.sucai999.com/).

In Equation (2), U represents the output voltage of the pressure sensor, unit: V. $U_{\text{0}}$ is the zero-drift voltage, unit: V. k represents the sensitivity. $k_{T}$ is the temperature coefficient of sensitivity. T and $T_{\text{0}}$ respectively represent the current ambient temperature and the calibration temperature of the pressure sensor (°C) [12]. Each component of the dynamics-based hardware system has a clear function, enabling basic pressure monitoring and control. However, it lacks monitoring for parameters such as flow, displacement, and oil temperature. Fixed-displacement pumps also consume high energy, exhibit limited adaptability to working conditions, and offer limited fault-diagnostic and precise-control capabilities [13]. To address the aforementioned issues, improvements were made by introducing flow sensors, displacement sensors, oil temperature sensors, cooling devices, and pressure compensation valves. As a result, the operational flowchart of the improved hardware system is shown in Fig 2.

Figure 2 adds flow sensors, displacement sensors, oil temperature sensors, cooling devices, and pressure-compensation valves based on Fig 1. This improved dynamics-based system draws oil from the tank and purifies it via a filter. An oil temperature sensor monitors temperature in real-time, and when the threshold is exceeded, the dynamic temperature control logic activates a cooling device. During oil delivery, a flow sensor monitors flow based on the dynamic model. The solenoid directional valve and pressure-compensating valve jointly regulate flow direction and pressure according to dynamic characteristics, driving the hydraulic cylinder. A displacement sensor simultaneously collects dynamic parameters of the piston stroke. Sensor data are fed into the control logic unit, which uses dynamic algorithms to identify anomalies and adjust the pump, valve, or cooling device. The oil returns to the tank, forming a cycle, and dynamic optimization ensures precise hydraulic control. The actual output power of the hydraulic pump is calculated as shown in Equation (3) [14].


$$P_{act}=\frac{p\times Q_{act}}{60\times \eta_{v}\times \eta_{m}}$$ (3)

In Equation (3), $P_{act}$ represents the actual output power of the hydraulic pump, reflecting its output capability, unit: W. $Q_{act}$ is the actual output flow, unit: m^3^/s. $\eta_{v}$ and $\eta_{m}$ represent volumetric and mechanical efficiencies, respectively. The oil delivered by the pump is further regulated by the solenoid directional valve. The calculation is shown in Equation (4) [15].


$$Q=C_{d}\cdot A_{v}\cdot \sqrt{\frac{2\Delta p}{\rho }}$$ (4)

**Fig 3:**
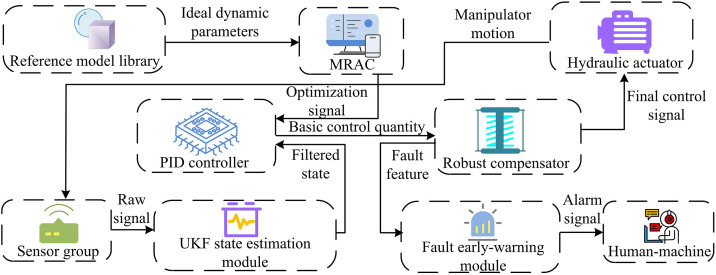
Structure of the MUP software system (Icon source from: https://icon.sucai999.com/).

In Equation (4), Q represents the flow rate through the valve, which determines oil speed, unit: m^3^/s. $A_{v}$ represents the valve port area, unit: m^2^, and Δp is the pressure difference across the valve, unit: Pa. Traditional software systems mostly use basic PID control algorithms with simple data display programs. These systems are low-cost and stable, making them suitable for regular conditions. However, they lack adaptive adjustment, require manual parameter tuning, and cannot handle nonlinear problems under complex conditions. Fault diagnosis also depends on manual inspection [16]. To further improve, MRAC and UKF intelligent algorithms were studied for optimization, and an MUP intelligent software system with self-optimization and fault warning functions was constructed. Its structure is shown in Fig 3.

As shown in Fig 3, the MUP system collects signals through sensor groups, processes them through the UKF state estimation module, fuses them with the ideal parameters output by the reference dynamic model, and inputs them into the MPC module to generate optimization signals. The PID controller outputs a signal to drive the robotic arm and provides real-time feedback on its status, enabling closed-loop, self-optimizing control based on dynamics. At the same time, the system extracts fault features and provides real-time alarms through the warning module to achieve the fault warning function. The feature extraction process mainly includes three parts. One is data preprocessing and alignment, which involves timestamp alignment and synchronization of multi-source heterogeneous time-series data from sensors such as pressure, displacement, flow rate, and temperature. Sliding time window slicing is used, and Z-score standardization is applied to eliminate dimensional differences. The second is spatiotemporal feature extraction, which inputs preprocessed multi-channel temporal data into a CNN and automatically extracts its temporal and spatial correlation features through multi-layer convolution and pooling operations. The third is dynamic state feature enhancement, which uses UKF to estimate system states such as pressure, position, and velocity in real-time. The estimated state vector is used as an enhancement feature and fused with the original signal features extracted by CNN to form a dynamic feature representation with physical meaning, which is used for subsequent fault diagnosis and control decision-making. The UKF state estimation is calculated as shown in Equation (5) [17].


$${\mathrm{\backslash stackrel}\wedge x}_{k|k-1}=f\left({\mathrm{\backslash stackrel}\wedge x}_{k-1|k-1},u_{k-1}\right)$$ (5)

In Equation (5), ${\mathrm{\backslash stackrel}\wedge x}_{k|k-1}$ represents the predicted state at time k based on the state at time k−1. $u_{k-1}$ is the control input vector at time k−1. The state update process is calculated as shown in Equation (6) [18].


$${\mathrm{\backslash stackrel}\wedge x}_{k|k}={\mathrm{\backslash stackrel}\wedge x}_{k|k-1}+K_{k}\left(z_{k}-h\left({\mathrm{\backslash stackrel}\wedge x}_{k|k-1}\right)\right)$$ (6)

**Fig 4:**
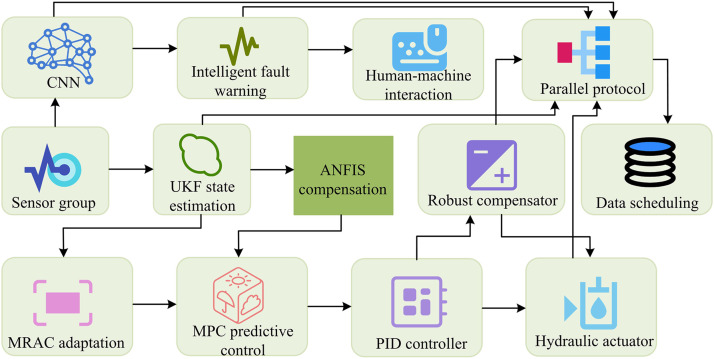
Operational flow of the SMUP software system (Icon source from: https://icon.sucai999.com/).

In Equation (6), ${\mathrm{\backslash stackrel}\wedge x}_{k|k}$ represents the updated state estimate at time k. $K_{k}$ is the UKF gain matrix, balancing prediction error and observation noise. $z_{k}$ is the raw signal collected by the sensor at time k, used for state estimation and control optimization. The MUP software system uses UKF for accurate state estimation. MPC and PID work together to enable self-optimization, combining predictability and real-time response. However, it still faces limitations in adapting to extreme conditions. Under complex interference, the model may be mismatched. The fault warning function also relies on preset thresholds and struggles to detect unknown faults [19]. To address this, ANFIS is introduced to adjust model parameters in real-time for extreme conditions. CNN is also introduced to extract fault features and identify unknown faults, enhancing fault warning capability. In addition, the study chose CNN instead of traditional shallow models such as Support Vector Machine (SVM) because the fault characteristics of hydraulic systems are often hidden in the spatiotemporal correlation of multi-source sensor data. Although SVM performs well in small sample classification, its kernel function needs to be manually designed, making it difficult to automatically extract deep spatiotemporal features. CNN can automatically learn discriminative feature representations from original vibration signals, pressure sequences, and other time-series data through convolutional and pooling layers. It is particularly adept at identifying early weak faults and composite fault patterns, which significantly improves the accuracy and generalization ability of fault diagnosis. Therefore, the study introduced ANFIS and CNN into the MUP software system to improve it and build the SMUP system. Its operational flow is shown in Fig 4.

As shown in Fig 4, the system first collects raw dynamic signals from the hydraulic system of the lifting manipulator wrist through sensor groups. Firstly, the UKF module is responsible for real-time estimation of the dynamic state of the hydraulic system. It provides accurate estimates of system status by processing raw data from sensors. These estimated values are used not only for current control decisions but also as input data for subsequent modules. The UKF output is directly transmitted to the ANFIS module, which uses these state estimates to optimize the control strategy and adapt to the hydraulic system’s dynamic characteristics. The ANFIS module ensures stability and accuracy of the system under complex operating conditions by adjusting control parameters. At the same time, the CNN module receives dynamic data from sensors, which is preprocessed and input into the CNN network. CNN extracts features from the data through convolution operations and identifies potential fault patterns. These fault characteristics are transmitted to the MPC module, which combines the state estimation of UKF and the fault characteristics of CNN for predictive control. The MPC module generates optimized control signals based on the predicted results, which are ultimately transmitted to the PID controller. The PID controller adjusts the actual operation of the hydraulic system based on these signals. Throughout the process, each module is coordinated through a central control logic unit. This control logic unit ensures the correct flow and processing of data and is also responsible for transmitting necessary control signals between modules. Through this tight coupling mechanism, the entire system can achieve efficient fault diagnosis and precise control. This integrated framework not only improves the overall performance of the system but also enhances its adaptability and reliability in complex operating conditions. The ANFIS compensation optimization is shown in Equation (7) [20].


$$y=\overset{M}{\underset{i=1}{\sum }}{\overset{―}{\omega }}_{i}\cdot f_{i}\left(x\right)$$ (7)

In Equation (7), ${\overset{―}{\omega }}_{i}$ represents the normalized weight of rule i. y is the final output of ANFIS used for control compensation. x is the multidimensional input vector. M is the predefined number of rules. The MRAC adaptive parameter adjustment is calculated as shown in Equation (8) [21].


$$\hat{\theta }=-\Gamma \cdot \phi \left(x\right)\cdot e^{T}\cdot P\cdot B$$ (8)

In Equation (8), $\hat{\theta }$ represents the control parameter vector. Γ is the adaptive gain matrix, which determines the adjustment speed and direction, directly affecting convergence, stability, and interference resistance. ϕ(x) is the regression vector. e is the tracking error.

### 2.2. Stability optimization for hydraulic controller of lifting manipulator wrist

**Fig 5:**
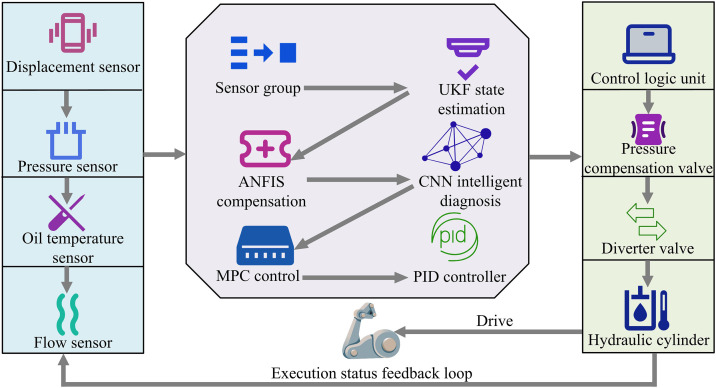
Operational flow of SH hydraulic controller model (Icon source from: https://icon.sucai999.com/).

The improved hardware system ensures precise, stable, and reliable hydraulic control by using a filter to keep the oil clean and various sensors to accurately collect status parameters. The improved software system uses intelligent algorithms to optimize trajectory control, monitor hardware status in real-time, and perform fault diagnosis. Therefore, this study integrates the hardware execution capability and software intelligent regulation to build the SH hydraulic controller model. The operational flow of this model is shown in Fig 5.

As shown in Fig 5, the model first collects real-time data from the hydraulic system using displacement, pressure, oil temperature, and flow sensors on the left. The data are passed to the central software algorithm processing unit. After the data are gathered, they pass through several modules—UKF state estimation, ANFIS compensation, CNN-based intelligent diagnosis, MPC model predictive control, and PID control—for deep integration and intelligent decision-making. The output control parameters are sent as control signals to the control logic unit in the hardware execution area on the right. Working with the pressure compensation valve and directional valve, it adjusts the cylinder’s movement and drives the manipulator wrist. Meanwhile, the actuator status is fed back to the sensors on the left, continuously updating the control instructions to ensure precise and safe lifting operations. The calculation for CNN-based intelligent diagnosis is shown in Equation (9).


$$\overset{l}{\underset{j}{x}}=\sum_{i\in M_{j}}\overset{l-1}{\underset{i}{x}}*\overset{l}{\underset{i,j}{k}}+\overset{l}{\underset{j}{b}}$$ (9)

In Equation (9), $\overset{l}{\underset{j}{x}}$ represents the j -th feature map in the l -th layer. M denotes the set of input feature maps, such as images converted from time-sequence sensor data. $\overset{l}{\underset{i,j}{k}}$ refers to the convolution kernel of the l -th layer. The calculation of MPC is shown in Equation (10) [22].


$${min}_{u_{k},...,u_{N-1}}\overset{N-1}{\underset{i=0}{\sum }}(\overset{\text{2}}{\underset{Q}{\|x_{k+i|k}-x_{ref}\|}}+\overset{\text{2}}{\underset{R}{\|u_{k+i}\|}})+\overset{\text{2}}{\underset{P}{\|x_{k+N|k}-x_{ref}\|}}$$ (10)

**Fig 6:**
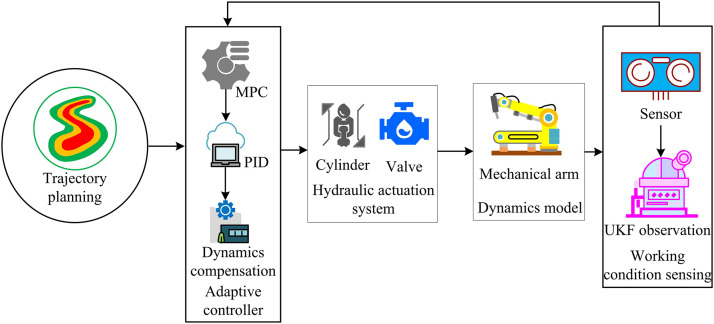
Structure of dynamics-based controller model (Icon source from: https://icon.sucai999.com/).

In Equation (10), N denotes the prediction horizon. $x_{ref}$ represents the reference state. Q, R, and P are the weight matrices. $u_{k+i}$ indicates the future control input sequence. However, it lacks consideration for dynamic characteristics such as force and inertia during manipulator movement. In complex working conditions, achieving precise adaptation is difficult, which affects system stability and operational efficiency. A dynamics-based controller can adjust the hydraulic output and control strategies in real-time, addressing the limitations of software–hardware integration in complex scenarios and improving overall system performance. Therefore, this study combines hardware and software with dynamics-based modeling to enhance system stability, improve efficiency, and ensure safe and precise execution of lifting tasks. The structure of the dynamics-based controller model is shown in Figure 6.

As shown in Fig 6, the model first generates the desired reference angles through the trajectory planning module. The adaptive controller receives these reference angles and simultaneously obtains real-time condition information from the environment. It then uses the dynamics model to compute suitable control commands. The hydraulic actuator system adjusts the hydraulic circuit based on these commands. During execution, the dynamics model continuously tracks changes in the manipulator and load, while the condition monitoring unit keeps checking the actual operating status. These feedback signals are sent back to the adaptive controller, which updates the control strategy to regulate the manipulator through the hydraulic system. The calculation of trajectory planning is shown in Equation (11).


$$\theta_{ref}\left(t\right)=a_{\text{0}}+a_{\text{1}}t+a_{\text{2}}t^{\text{2}}+a_{\text{3}}t^{\text{3}}$$ (11)

In Equation (11), $\theta_{ref}\left(t\right)$ indicates the desired position angle of the joint or end-effector of the manipulator at time t, unit: rad. After trajectory planning and status information collection, the dynamics model performs further calculation as shown in Equation (12).


$$M\left(\theta \right)\ddot{\theta }+C\left(\theta ,\dot{\theta }\right)\dot{\theta }+G\left(\theta \right)=\tau$$ (12)

**Fig 7:**
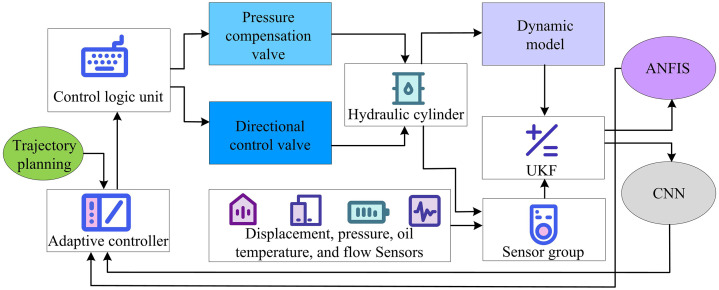
Operational flow of SHD controller (Icon source from: https://icon.sucai999.com/).

In Equation (12), M(θ) represents the inertia matrix, reflecting system inertia, unit: kg· m^2^. $C\left(\theta ,\dot{\theta }\right)$ is the centrifugal matrix, unit: kg· m^2^. G(θ) is the gravity vector, unit: N· m. θ, $\dot{\theta }$, and $\ddot{\theta }$ respectively represent joint angle, angular velocity, and angular acceleration, all in rad/s^2^. M(θ) dynamically incorporates changes in load mass and inertia tensor through an online estimation mechanism, accurately reflecting the inertia characteristics of the system when grasping different loads. $C\left(\theta ,\dot{\theta }\right)$ describes the nonlinear force effects generated by the coupling of joint movements, providing a key theoretical basis for designing passive control laws and proving system stability. G(θ) calculates based on the DH parameters and center of gravity position of the robotic arm, and also includes the contribution of load moment, enabling the controller to accurately compensate for gravity moment fluctuations caused by arm posture and load changes. This dynamic model is not a fixed theoretical framework, but is called in real-time as the core computing module of the SHD controller. During each control cycle, the adaptive controller dynamically calculates the current M(θ), $C\left(\theta ,\dot{\theta }\right)$, and G(θ) based on real-time state estimation from the sensor group provided by UKF, thereby generating feedforward control torque that can accurately counteract nonlinear dynamic effects. Finally, the SH model and the dynamics-based controller model are integrated to construct the SHD hydraulic controller for the lifting manipulator wrist. The operational flow is shown in Fig 7.

As shown in Fig 7, the model first generates the desired manipulator motion trajectory using the trajectory planning module. The trajectory data are sent to the adaptive controller. Meanwhile, a sensor group consisting of displacement and pressure sensors collects real-time system data and sends it to the UKF module for state estimation. The processed data is used by the dynamics model to analyze the manipulator’s dynamic characteristics. It is also combined with ANFIS and CNN for deep data fusion and intelligent fault diagnosis. The adaptive controller integrates the trajectory plan and processed results to issue commands to the control logic unit. The control logic drives the pressure compensation valve and directional valve, adjusts the hydraulic cylinder, and activates the manipulator. Cylinder status is fed back to the sensor group to form a closed loop, ensuring precise and stable hydraulic control. The calculation of the oil temperature sensor is shown in Equation (13).


$$\left\{ \begin{array}{c} @l@R_{T}=R_{T_{\text{0}}}\cdot exp\left[B(\frac{\text{1}}{T}-\frac{\text{1}}{T_{\text{0}}})\right]\\ T=\frac{\text{1}}{\frac{\text{1}}{T_{\text{0}}}+\frac{\text{1}}{B}ln\left(\frac{R_{T}}{R_{T_{\text{0}}}}\right)} \end{array}\right.$$ (13)

In Equation (13), $R_{T}$ represents the resistance value of the thermistor at oil temperature T, unit: Ω. Similarly, $R_{T_{\text{0}}}$ denotes the resistance at reference temperature $T_{\text{0}}$,unit: Ω, and T indicates the oil temperature,unit: °C. The displacement sensor calculation is shown in Equation (14).


$$x=x_{\text{0}}+\int vdt$$ (14)

In Equation (14), x is the current displacement of the piston, measured as the linear distance from the initial position $x_{\text{0}}$. v represents the piston velocity. The adaptive control calculation is shown in Equation (15).


$$u=K_{p}e_{x}+K_{d}\frac{d_{e_{x}}}{dt}+K_{i}\int e_{x}dt$$ (15)

In Equation (15), u denotes the control output command. $K_{p}$, $K_{d}$, and $K_{i}$ are the proportional, derivative, and integral gains adjusted adaptively.

## 3. Experimental results

### 3.1 Parameter settings and implementation details

To verify the superiority of the SHD hydraulic controller, this study compared it with three other controllers: the QINN-RC controller combining Quantum-Inspired Neural Network (QINN) and Robust Control (RC), the ML-FWC controller combining Meta-Learning (ML) and Fuzzy Wavelet Control (FWC), and the FL-EC controller combining Federated Learning (FL) and Edge Control (EC). The QINN-RC controller leverages the superposition states of quantum bits to optimize the weight distribution of hidden nodes in neural networks; ML-FWC adopts a two-layer learning framework, with the inner layer using a fuzzy wavelet network as the base controller and the outer layer introducing a meta learner. FL-EC trains local controllers for each robotic arm on local data, performs real-time control tasks, and uses the FL framework to periodically aggregate model parameters from all edge devices. The updated global model is then distributed to each edge device to improve the performance of each individual controller. For fair comparison, all models were tested on a unified hardware platform and dataset. In addition, the parameter settings of the comparative model are as follows: the quantum layer number and superposition state parameters of QINN-RC are set to 4 and 0.5, respectively, and the gain parameter is set to 2.5; The learning rates of the inner and outer loops of ML-FWC are set to 0.01 and 0.001, respectively, and the fuzzy wavelet network contains 25 rules. FL-EC conducts 50 rounds of federated training at a client sampling rate of 0.8, with local training cycles and batch sizes set to 5 and 32, respectively. All hyperparameters of the models were optimized and determined on the validation set through grid search, ensuring the rigor of the comparative experiments and the reproducibility of the results. The specific simulation process was as follows: Equations (1), (2), (3), (4), (13) and (14) described the physical principles of hydraulic systems, which are used to construct high-fidelity simulation models. Equations (5)–(10) define the SHD controller’s intelligent core. During simulation and actual operation, these algorithms were encoded and embedded in the control loop. The UKF algorithm estimated the system state in real-time, provided input for MPC and ANFIS; Nonlinear and parameter uncertainties of the ANFIS and MRAC online compensation systems; CNN processed sensor data streams for fault feature extraction; MP performed rolling optimization prediction, and the final generated control variable is output through adaptive PID, i.e., Equation (15). Equation (2) was used to understand the output characteristics of pressure sensors, in order to ensure signal accuracy when designing data acquisition systems. The dynamic models of Equations (11) and (12) provided a theoretical framework for analyzing trajectory tracking errors.

The experimental dataset was obtained from a real lifting manipulator operated by a heavy industry company. The data included hydraulic system operation over 500 hours, covering 12 working conditions such as no-load and full-load, and included eight fault scenarios such as pipeline leakage and valve sticking. At the same time, the data acquisition system is built on the NI cDAQ-9188 Ethernet chassis and the NI 9234 analog input module to ensure high-precision synchronous sampling. The sampling frequency is uniformly set to 1000 Hz to meet the requirements of capturing high-frequency dynamic characteristics of hydraulic systems. The collected sensor signals include: MikroPiranha CMP331 pressure sensor for measuring hydraulic cylinder pressure, MTS Temposonics RH series magnetostrictive displacement sensor for detecting piston displacement, and IFM SM9000 temperature and flow composite sensor for monitoring oil state. The hardware configuration of the experimental control platform is an industrial server equipped with an Intel Xeon Gold 6248R processor and 128 GB of DDR4 memory, and an NVIDIA RTX A6000 graphics card to accelerate the training and inference of deep learning modules such as CNNs. Real-time control tasks run on the Ubuntu 20.04 operating system, achieving deterministic real-time control through the MathWorks xPC Target environment, with a strictly locked control cycle of 2ms. All comparison algorithms are implemented within the PyTorch 1.13 framework, ensuring high precision and synchronization throughout the entire process, from data acquisition and algorithm processing to control execution. In addition, the dataset contains the following multi-source heterogeneous data signals: motion state signals of joint angle, angular velocity, and end displacement; Hydraulic system signals for pump outlet pressure, cylinder chamber pressure, oil temperature, and flow rate values; Control signals for valve-controlled voltage, PID output, and trajectory commands; Fault injection signal. The experimental inputs include joint angle trajectory commands, load torque commands, and simulated environmental disturbances. The output indicators are key performance parameters such as system evaluation trajectory tracking error, pressure control accuracy, fault detection accuracy, system response time, and energy conversion efficiency. The specific data preprocessing process is as follows: first, timestamp alignment is performed on sensor data with different sampling rates, and slices are performed within a 0.5-second window, resulting in over 3.6 million valid data samples; Secondly, the 3 σ criterion is used to remove obvious abnormal data points, and linear interpolation is used to fill in missing values. Then perform Z-score standardization on the data to eliminate the influence of dimensionality; Finally, the training set, validation set, and testing set are randomly divided in a ratio of 7:2:1 to ensure that each operating condition and fault type is evenly distributed in the three datasets.

The experimental parameters are set as follows: CNN uses 4 convolutional layers and 2 fully connected layers. The number of channels in the convolutional layers is 32, 64, 128, and 128, respectively. At the same time, a linear rectification unit activation function (RELU) is used. After each convolutional layer, a 2 × 1 max pooling layer is added for dimensionality reduction. After flattening the feature map, it is fed into two fully connected layers with 128 and 64 neurons, respectively. Finally, a Softmax output layer is used to classify 8 fault types. The network is trained using the Adam optimizer with an initial learning rate of 0.001, batch size set to 128, and iteration count set to 100.

### 3.2. Comparative experimental analysis of SHD hydraulic controller

**Fig 8:**
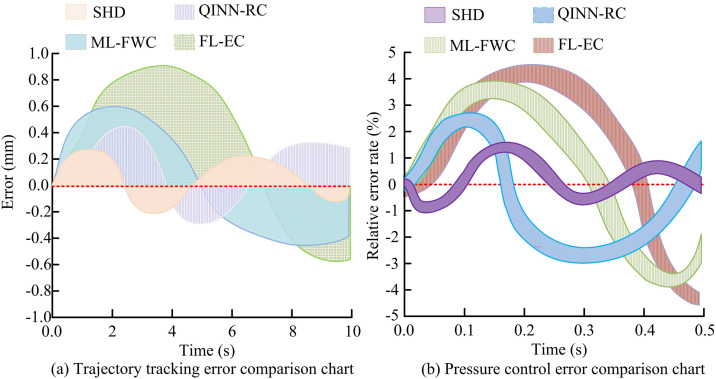
Comparison of trajectory tracking error and pressure control error.

The trajectory-tracking and pressure-control errors for the four controllers are shown in Fig 8.

**Fig 9:**
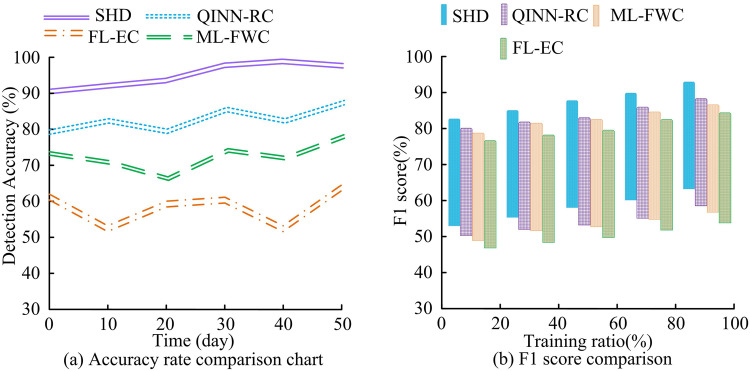
Comparison of fault detection accuracy and F1 score.

As shown in Fig 8(a), the SHD controller had the smallest fluctuation in trajectory tracking error, with a maximum error of only 0.28 mm. The QINN-RC controller followed, with a maximum error of 0.45 mm. The ML-FWC and FL-EC controllers had larger errors, with the FL-EC controller showing the worst performance, having a maximum error of 0.90 mm. According to Fig 8(b), the SHD controller had a maximum relative pressure control error of 1.5%. The maximum relative errors of QINN-RC, ML-FWC, and FL-EC were −2.9%, 3.7%, and 4.6%, respectively. This confirms that the research method significantly surpasses existing advanced methods in trajectory tracking and pressure control accuracy, effectively solving the problem of insufficient accuracy of existing control methods in complex working conditions. Next, this study tested the fault detection accuracy and F1 score of the four controllers. The results are shown in Fig 9.

**Fig 10:**
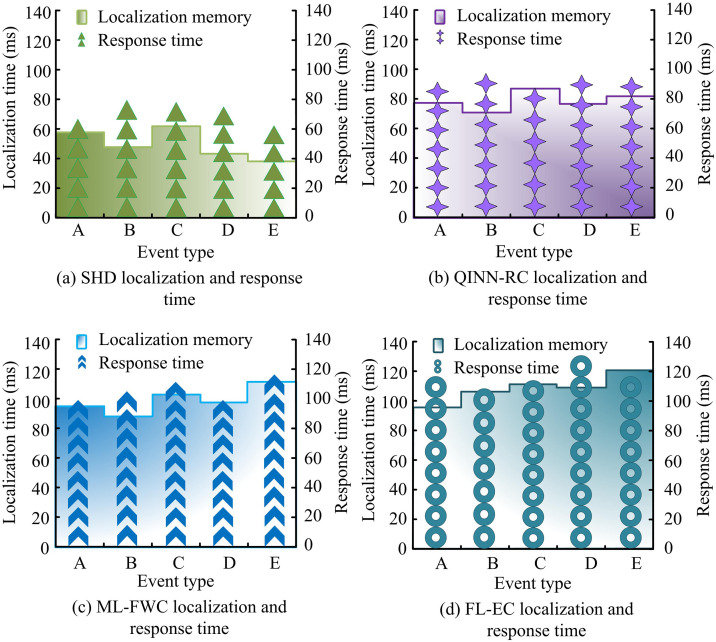
Comparison of fault location time and safety protection response time.

The comparison of fault detection accuracy rates shown in Fig 9(a) indicates that the SHD controller maintained a stable high level during the 50-day test without significant fluctuations, with the highest detection accuracy rate reaching 98.3%. This is attributed to the deep feature extraction capability of its CNN module for multi-source dynamic data, as well as the robust state estimation and parameter adaptation jointly provided by UKF and ANFIS. In contrast, the QINN-RC and ML-FWC controllers showed a downward trend in the middle and later stages of the test. Especially when facing compound faults and sudden changes in working conditions, their recognition ability was insufficient, reflecting the limited adaptability of their models to dynamic environmental changes. The accuracy rate of the FL-EC controller has always been relatively low, indicating that under the distributed learning framework, local data heterogeneity and communication delay have significantly affected its fault diagnosis performance. The F1 value in Fig 9(b) further reveals the comprehensive capabilities of each controller. The SHD controller leads significantly with an F1 value of 94.3%, indicating its excellent performance in reducing false alarms and missed alarms. This is mainly attributed to the collaborative mechanism between CNN and MPC: CNN accurately identifies fault features, while MPC adjusts the control strategy based on the predicted information to avoid unnecessary system responses caused by misjudgment of faults. The gap in F1 value between QINN-RC and ML-FWC, especially the insufficient identification of fault types with significant dynamic characteristics such as abnormal flow control, highlights its structural limitations in spatio-temporal feature fusion and decision collaboration. The above results verify the effectiveness and reliability of the SHD controller in handling complex scenarios, such as multiple concurrent faults and time-varying working conditions in the hydraulic system. To further verify the safety response efficiency, this study compared the fault location time and safety protection response time of the four controllers under five hazardous events: overload, over-temperature, sudden pressure drop, abnormal vibration, and abnormal liquid level, labeled as A, B, C, D, and E, respectively. The results are shown in Fig 10.

**Fig 11:**
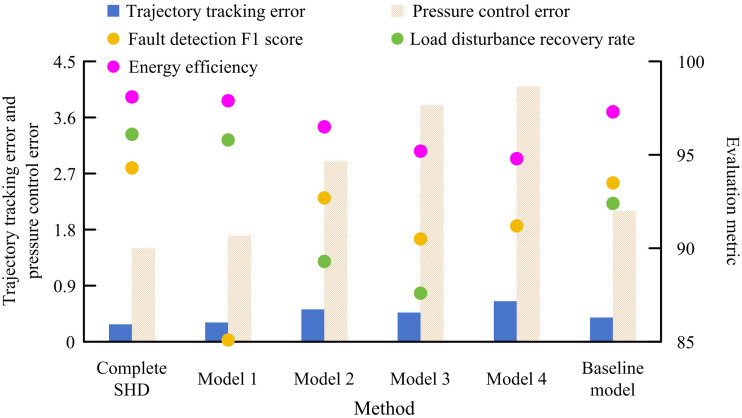
Results of the ablation experiment.

As shown in Fig 10(a), the SHD controller achieved a maximum fault location time of 65 ms and a maximum safety response time of 79 ms. According to Fig 10(b), the QINN-RC controller’s maximum fault location time and safety response time were 89 ms and 98 ms, respectively. Figs 10(c) and 10(d) showed that the ML-FWC controller reached 112 ms and 119 ms, while the FL-EC controller recorded the highest delays at 121 ms and 129 ms. This result indicates that the SHD controller significantly reduces the delay from fault occurrence to safety intervention, effectively solving the problem of response lag in existing methods when dealing with sudden working conditions. Finally, ablation experiments were conducted to further explore the contribution of each key algorithm module to the final performance. The specific settings are as follows: Model 1: Using traditional threshold rules instead of CNN for fault diagnosis; Model 2: Disable adaptive neural fuzzy compensator; Model 3: Directly using the raw sensor readings as the system state; Model 4: Cancel the feedforward torque calculation based on the dynamic model; Benchmark model: Directly use PID controller. The result can be obtained as shown in Fig 11.

As shown in Fig 11, when using Model 1, the fault detection F1 score decreased from 94.3% to 85.1%, demonstrating that the intelligent diagnosis method based on deep learning has significant advantages in extracting complex fault features. Model 2 resulted in an 85.7% increase in trajectory tracking error, rising from 0.28 mm to 0.52 mm, while the load disturbance recovery rate decreased by 7.1%, demonstrating the critical value of this module in handling system nonlinearity. The pressure control error of Model 3 increased by 153%, from 1.5% to 3.8%, indicating that accurate state estimation is the fundamental guarantee for control accuracy. The performance degradation brought by Model 4 is the most significant, with a 132% increase in trajectory tracking error and a 12.1% decrease in load disturbance recovery rate, verifying that model-based feedforward control is the core for dealing with complex dynamics. The direct use of a PID controller in the benchmark model did not cause system collapse, but still increased trajectory tracking error by 39%, proving that the introduction of advanced control algorithms has important value in improving system dynamic performance. These results fully confirm the unique value of each module in the SHD controller and the effectiveness of its collaborative working mechanism, providing strong experimental evidence for control strategies based on multi-algorithm fusion.

### 3.3. Performance and stability analysis of SHD hydraulic controller

**Fig 12:**
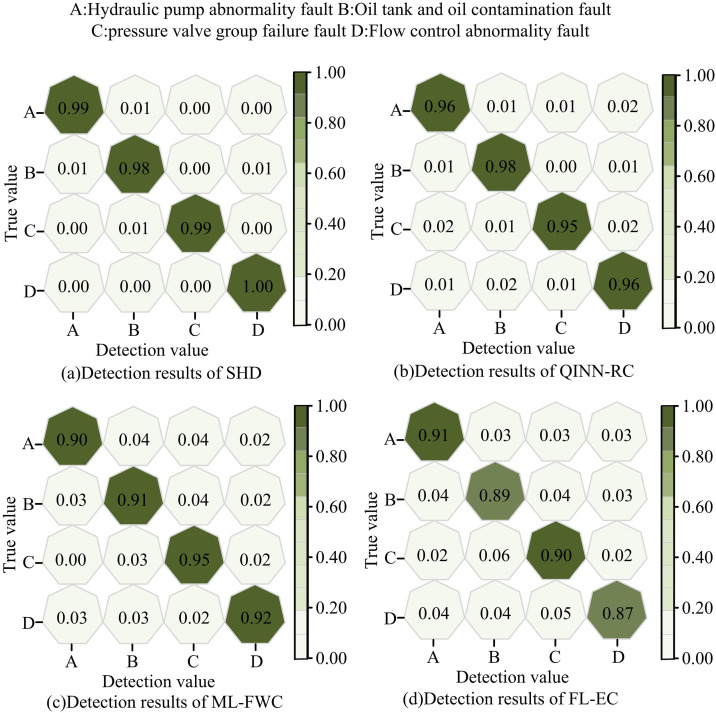
Comparison of fault classification accuracy.

After validating the performance of the SHD hydraulic controller, this study further evaluated its capabilities by comparing it with the QINN-RC, ML-FWC, and FL-EC controllers. The experimental platform consisted of a high-precision data acquisition system, a working condition simulation device, an intelligent control terminal, and a visualization analysis platform. The dataset used followed the guidelines of ISO 19978:2017, the international standard for load-pressure characteristic testing of hydraulic manipulators. This study classified and identified four types of faults using the SHD, QINN-RC, ML-FWC, and FL-EC controllers: hydraulic pump faults, oil tank and fluid contamination faults, pressure valve group failures, and flow control anomalies. The classification accuracy results are shown in Fig 12.

**Fig 13:**
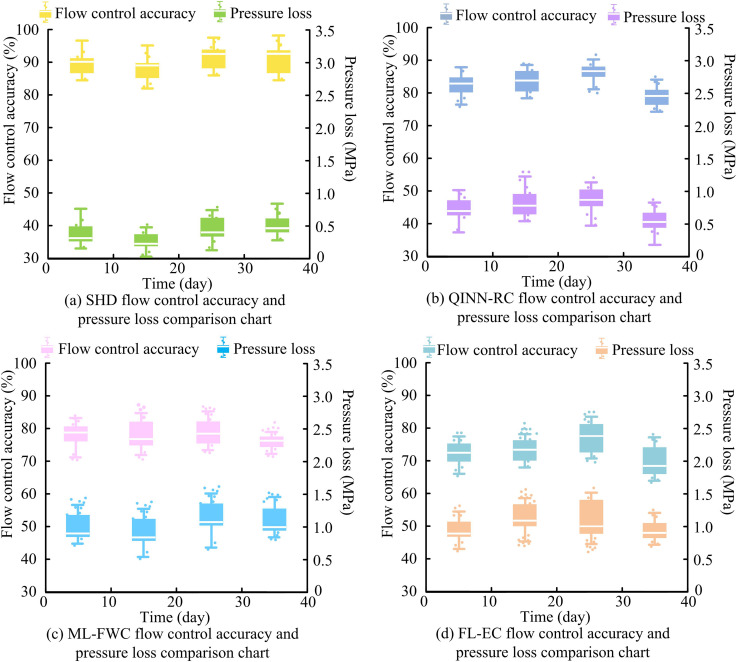
Comparison of flow control accuracy and pressure loss.

As shown in Fig 12(a), the SHD controller achieved the highest classification accuracy of 100.0% for identifying flow control anomalies, and its lowest accuracy was 98.0%. Figure 12(b) showed that the QINN-RC controller reached a maximum accuracy of 98.0% and a minimum of 95.0%. Figure 12(c) and 12(d) indicated that the highest classification accuracies of the ML-FWC and FL-EC controllers were 95.0% and 91.0%, respectively. These results demonstrated that the SHD controller achieved the highest accuracy in fault classification. The reason for achieving a recognition accuracy of 100.0% was that the dataset used for training and testing contained numerous high-quality data points, combined with the powerful nonlinear feature extraction capabilities of the CNN, which enabled the model to fully learn the precise patterns of the four fault types and achieve perfect classification. To evaluate the hydraulic performance of the four controllers, this study compared their flow control accuracy and pressure loss. The results are shown in Fig 13.

**Fig 14:**
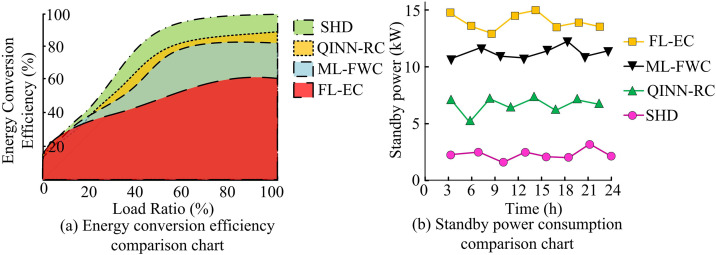
Comparison of energy conversion efficiency and standby power consumption.

As shown in Fig 13(a), the SHD controller achieved the highest flow control accuracy at 98.3% and the lowest maximum pressure loss at 0.71 MPa. Figure 13(b) showed that the QINN-RC controller had a maximum flow accuracy of 93.2% and a maximum pressure loss of 1.37 MPa. According to Fig 13(c), the ML-FWC controller reached a maximum flow control accuracy of 86.3%, with a maximum loss of 1.74 MPa. Figure 13(d) indicated that the FL-EC controller performed the worst, with a maximum accuracy of 84.5% and a maximum pressure loss of 1.84 MPa. These results demonstrated that the SHD controller maintained high control accuracy while minimizing pressure loss. Next, to evaluate energy performance, this study compared the energy conversion efficiency and standby power consumption of the four controllers. The results are shown in Fig 14.

As shown in Fig 14(a), the SHD controller achieved the highest energy conversion efficiency, reaching 98.1%, which significantly outperformed the other controllers. Figure 14(b) showed that the SHD controller had the lowest maximum standby power consumption at only 6.1 kW, while the QINN-RC, ML-FWC, and FL-EC controllers reached maximum standby consumption levels of 9.4 kW, 12.8 kW, and 14.7 kW, respectively. These findings demonstrated that the SHD controller offered high energy efficiency with low standby power consumption. Finally, to verify stability, this study compared the four controllers across five metrics: static error rate, overshoot, gain margin, load disturbance recovery rate, and nonlinear output deviation percentage. The static error rate is the ratio of the difference between the system’s actual and expected outputs under steady-state conditions, reflecting the controller’s accuracy over the long term. In the experiment, the static error rate was calculated by measuring the actual output value multiple times and comparing it with the expected output value. The overshoot reflects the stability and response speed of the system in dynamic response, calculated as follows, $CT=\left[\left(X_{max}-\overset{―}{X}\right)/\overset{―}{X}\right]\times 100$. Among them, $X_{max}$ and $\overset{―}{X}$ are the maximum response value and expected value, respectively. Stability margin referred to the gain or phase margin of a system at the stability boundary, reflecting the stability of the system when parameters changed. The calculation is as follows, $WH=20{log}_{10}\left(1/zh\right)$, zh indicates gain margin. The load interference recovery rate reflects the adaptability and recovery ability of the system in the face of external interference. The calculation is as follows: $GH=\left[\left(Y_{h}-Y_{g}\right)/Y_{g}\right]\times 100$, $Y_{h}$ and $Y_{g}$ are the outputs after recovery and interference, respectively. Nonlinear output deviation percentage refers to the ratio of deviation between the actual output and the expected output of a system under nonlinear conditions, which reflected the stability and accuracy of the system under nonlinear conditions. The results are shown in Table 1.

**Table 1:** Comparison of static error rate, overshoot, gain margin, and other indicators.

Types of stability	SHD	QINN-RC	ML-FWC	FL-EC
Steady-state error rate (%)	3.1	5.8	9.4	11.5
Overshoot (%)	1.2	4.6	7.9	8.7
Stability margin (dB)	12.4	10.3	7.2	6.1
Load disturbance recovery rate (%)	96.1	90.3	82.1	80.5
Percentage of output deviation caused by nonlinearity (%)	4.1	5.9	7.8	11.1

As shown in Table 1, the SHD controller achieved a static error rate of 3.1%, an overshoot of 1.2%, a gain margin of 12.4 dB, a load disturbance recovery rate of 96.1%, and a nonlinear output deviation of 4.1%. For the QINN-RC controller, the static error rate, overshoot, gain margin, recovery rate, and output deviation were 5.8%, 4.6%, 10.3 dB, 90.3%, and 5.9%, respectively. The ML-FWC and FL-EC controllers showed weaker performance, with static error rates of 9.4% and 11.5%, and gain margins of only 7.2 dB and 6.1 dB, respectively. These results confirmed that the SHD controller demonstrated superior stability in terms of static, dynamic, robust, load-resistant, and nonlinear hydraulic conditions.

Finally, to rigorously evaluate the model’s generalization ability, an additional 5-fold cross-validation was conducted. The results show that the SHD controller has an average recognition accuracy of 99.7% and a standard deviation of 0.15% for faults such as “abnormal flow control”. This indicates that the 100.0% accuracy in the above results is not a result of chance or overfitting, but rather a reflection of the model’s strong and stable discriminative ability in various fault features. The reason why this fault can be perfectly identified is that the flow signal attenuation pattern it causes is very unique and fully characterized in the dataset.

## 4. Discussion

The experimental results comprehensively verified the superior performance of the SHD controller architecture. The maximum trajectory tracking error is 0.28 mm, indicating that the control accuracy of the hydraulic manipulator under dynamic loading conditions has been significantly improved. This level of precision is basically achieved by a real-time dynamic model, which calculates the joint-specific inertia and Coriolis force, allowing the controller to predict and compensate for nonlinear coupling. This active compensation mechanism confirms that the system can still maintain the optimal hydraulic pressure under rapid load changes, with a pressure control error as low as 1.5%.

The fault detection subsystem demonstrated remarkable robustness, achieving an accuracy rate of 98.3% in identifying various fault conditions. This performance stems from the hierarchical data processing architecture, in which UKF provides filtering-state estimation for the CNN to extract deep features. The convolutional layer of CNN effectively captures the spatiotemporal features of early faults, such as valve viscosity and pipeline leakage, which typically present as subtle patterns in pressure and flow time series data [23–24]. The 100.0% accuracy rate of abnormal flow control classification particularly highlights the model’s ability to distinguish between normal operational changes and true fault conditions, which is a key capability to prevent false alarms in industrial environments.

The energy efficiency index reflects the best performance of the controller under continuous operation. The 98.1% energy conversion efficiency and 6.1kW standby power consumption demonstrate the synergistic effect of MPC predictive optimization and real-time regulation of pressure compensation valves. By minimizing throttling losses and optimizing pump displacement, this system reduces the typical 12–15% energy waste of traditional hydraulic systems. Given the increasing emphasis on sustainable industrial operations, this efficiency improvement is particularly remarkable [25–26].

The stability analysis confirmed the robustness of the controller in multiple operational dimensions. A static error rate of 3.1% and an overshoot rate of 1.2% demonstrate excellent steady-state and transient performance, while a gain margin of 12.4 dB provides strong robustness against parameter variations. The 96.1% load disturbance recovery rate demonstrates the system’s ability to maintain performance under external disturbances, which is a key requirement for applications involving variable payloads. These stability characteristics originate from an adaptive control framework that continuously adjusts PID gain based on real-time dynamic conditions [27–28].

The safe response time for fault location is 65 ms, and for protection activation, it is 79 ms, setting a new benchmark for hydraulic system safety. This rapid response is achieved through a parallel processing architecture, in which fault diagnosis and control adjustment occur simultaneously rather than sequentially. The ability of this system to maintain control accuracy while implementing security protocols represents a significant advancement in traditional methods, where security interventions typically undermine operational continuity [29–30].

Compared with existing hybrid intelligent controllers, the research method has the following unique contributions: firstly, unlike reinforcement learning based controllers such as RL-MPC and QINN-RC that rely on empirical exploration, the research method provides physical constraint guidance through dynamic models, avoiding training instability problems and possessing high-precision control capabilities in the initial stage. Secondly, compared to traditional adaptive robust control methods such as ARC and FL-EC, the research method introduces CNN for fault feature extraction and UKF for state estimation, achieving active perception and compensation for unmodeled dynamics and sudden faults. Finally, through the collaboration of ANFIS and MRAC, the system can still maintain stability in complex scenarios where parameter uncertainty, nonlinear friction, and external disturbances coexist. Its trajectory tracking accuracy is 0.28 mm, fault recognition rate is 100%, pressure control error is 1.5%, and other key indicators are superior to the comparative model, verifying its comprehensive advantages in dynamic load and multi-fault concurrent scenarios.

## 5. Conclusion

A SHD controller was proposed in this study. The experimental results showed that the controller achieved a maximum trajectory tracking error of 0.28 mm, a flow control accuracy of 98.3%, an energy conversion efficiency of 98.1%, a system steady-state error rate of 3.1%, and an overshoot of 1.2%. It significantly outperformed the comparison models in all key indicators, effectively addressing the problems of response delay and insufficient control accuracy of existing hydraulic control methods under complex working conditions. This research provided innovative methods and technical support for improving the operational accuracy, safety, and energy efficiency of hydraulic robotic arms. However, this study still has deficiencies, including cross-scenario adaptability, lightweight controller design, and edge deployment. Therefore, future research can be carried out on the following aspects. On the one hand, develop differentiated controllers for typical industrial scenarios, and adopt lightweight design and edge deployment solutions to reduce reliance on centralized computing and enhance the real-time performance and reliability of the system in resource-constrained environments. On the other hand, it is necessary to explore digital twins and online learning mechanisms to achieve continuous self-optimization and dynamic adaptation of the controller, and promote the evolution of the hydraulic control system towards a higher degree of autonomous intelligence.

## Supporting information

S1 File Minimal data set definition. (DOCX)
