# Supplementary material for: Design of a dynamics-based hydraulic controller for lifting manipulator wrist and its stability analysis
Source: PLoS One. 2026 Apr 28;21(4):e0347838. doi: 10.1371/journal.pone.0347838 (PMC13124004; doi:10.1371/journal.pone.0347838)
Supplement: S1 File — (DOCX) [file pone.0347838.s001.docx]

Minimal Data Set Definition

**The data in Figure 8**

| Evaluation category | Time (s) | SHD | QINN-RC | ML-FWC | FL-EC |
| --- | --- | --- | --- | --- | --- |
| Trajectory tracking error | 0 | 0.00 | 0.00 | 0.00 | 0.00 |
|  | 2 | 0.10 | 0.27 | 0.31 | 0.24 |
|  | 4 | -0.18 | -0.24 | 0.36 | -0.22 |
|  | 6 | 0.21 | -0.32 | -0.33 | 0.26 |
|  | 8 | 0.08 | 0.35 | -0.52 | -0.43 |
|  | 10 | -0.10 | 0.26 | -0.43 | -0.58 |
| Pressure control error | 0.0 | 0.00 | 0.00 | 0.00 | 0.00 |
|  | 0.1 | -0.24 | 2.03 | 2.87 | 1.73 |
|  | 0.2 | 1.07 | -2.46 | 3.25 | 3.81 |
|  | 0.3 | -0.75 | -2.95 | 1.26 | 3.24 |
|  | 0.4 | 0.24 | -2.47 | -3.51 | 0.52 |
|  | 0.5 | 0.00 | 0.62 | -3.02 | -4.32 |

**The data in Figure 9**

| Evaluation category | Time (day) | 0 | 10 | 20 | 30 | 40 | 50 |
| --- | --- | --- | --- | --- | --- | --- | --- |
| Detection accuracy (%) | SHD | 92.3 | 93.6 | 94.2 | 98.3 | 98.0 | 97.8 |
|  | QINN-RC | 78.6 | 84.5 | 77.3 | 87.4 | 85.2 | 88.9 |
|  | ML-FWC | 72.4 | 68.3 | 65.6 | 73.5 | 71.1 | 78.7 |
|  | FL-EC | 61.5 | 52.3 | 59.7 | 60.4 | 52.6 | 66.9 |
| F1 score (%) | Training ratio (%) | 0 | 20 | 40 | 60 | 80 | 100 |
|  | SHD | / | 82.6 | 85.4 | 87.9 | 93.1 | 94.3 |
|  | QINN-RC | / | 79.6 | 80.3 | 81.7 | 85.4 | 88.2 |
|  | ML-FWC | / | 78.2 | 80.1 | 80.9 | 84.2 | 85.3 |
|  | FL-EC | / | 77.1 | 77.8 | 78.2 | 80.2 | 81.6 |

**The data in Figure 10**

| Method | Evaluation index | A | B | C | D | E |
| --- | --- | --- | --- | --- | --- | --- |
| SHD | Localization time (ms) | 59 | 48 | 65 | 40 | 36 |
|  | Response time (ms) | 64 | 79 | 76 | 75 | 63 |
| QINN-RC | Localization time (ms) | 79 | 72 | 88 | 78 | 82 |
|  | Response time (ms) | 92 | 98 | 88 | 97 | 96 |
| ML-FWC | Localization time (ms) | 94 | 88 | 102 | 95 | 112 |
|  | Response time (ms) | 99 | 103 | 112 | 95 | 118 |
| FL-EC | Localization time (ms) | 96 | 108 | 110 | 109 | 124 |
|  | Response time (ms) | 118 | 108 | 110 | 130 | 116 |

**The data in Figure 11**

| Method | Trajectory tracking error (mm) | Pressure control error (%) | Fault detection F1 score (%) | Load disturbance recovery rate (%) | Energy efficiency (%) |
| --- | --- | --- | --- | --- | --- |
| Complete SHD controller | 0.28 | 1.5 | 94.3 | 96.1 | 98.1 |
| Model 1 | 0.31 | 1.7 | 85.1 | 95.8 | 97.9 |
| Model 2 | 0.52 | 2.9 | 92.7 | 89.3 | 96.5 |
| Model 3 | 0.47 | 3.8 | 90.5 | 87.6 | 95.2 |
| Model 4 | 0.65 | 4.1 | 91.2 | 84.5 | 94.8 |
| Baseline model | 0.39 | 2.1 | 93.5 | 92.4 | 97.3 |

**The data in Figure 12**

| Method | Detection value | True value | | | |
| --- | --- | --- | --- | --- | --- |
|  |  | A | B | C | D |
| SHD | A | 0.99 | 0.01 | 0.00 | 0.00 |
|  | B | 0.01 | 0.98 | 0.00 | 0.01 |
|  | C | 0.00 | 0.01 | 0.99 | 0.00 |
|  | D | 0.00 | 0.00 | 0.00 | 1.00 |
| QINN-RC | A | 0.96 | 0.01 | 0.01 | 0.02 |
|  | B | 0.01 | 0.98 | 0.00 | 0.01 |
|  | C | 0.02 | 0.01 | 0.95 | 0.02 |
|  | D | 0.01 | 0.02 | 0.01 | 0.96 |
| ML-FWC | A | 0.90 | 0.04 | 0.04 | 0.02 |
|  | B | 0.03 | 0.91 | 0.04 | 0.02 |
|  | C | 0.00 | 0.03 | 0.95 | 0.02 |
|  | D | 0.03 | 0.03 | 0.02 | 0.92 |
| FL-EC | A | 0.91 | 0.03 | 0.03 | 0.03 |
|  | B | 0.04 | 0.89 | 0.04 | 0.03 |
|  | C | 0.02 | 0.06 | 0.90 | 0.02 |
|  | D | 0.04 | 0.04 | 0.05 | 0.87 |

**The data in Figure 13**

| Method | Evaluation index | 10 | 20 | 30 | 40 |
| --- | --- | --- | --- | --- | --- |
| SHD | Flow control accuracy (%) | 97.80 | 98.00 | 98.30 | 97.90 |
|  | Pressure loss (MPa) | 0.52 | 0.59 | 0.63 | 0.62 |
| QINN-RC | Flow control accuracy (%) | 83.00 | 81.50 | 87.20 | 78.40 |
|  | Pressure loss (MPa) | 0.70 | 0.72 | 0.74 | 0.69 |
| ML-FWC | Flow control accuracy (%) | 78.50 | 76.80 | 77.30 | 76.50 |
|  | Pressure loss (MPa) | 1.15 | 1.13 | 1.24 | 1.22 |
| FL-EC | Flow control accuracy (%) | 74.00 | 73.20 | 78.50 | 68.80 |
|  | Pressure loss (MPa) | 0.85 | 1.21 | 1.03 | 0.97 |

**The data in Figure 14**

| Evaluation category | Load Ratio (%) or Time (h) | SHD | QINN-RC | ML-FWC | FL-EC |
| --- | --- | --- | --- | --- | --- |
| Energy Conversion Efficiency (%) | 0 | 18.8 | 18.6 | 18.5 | 18.2 |
|  | 20 | 30.5 | 29.4 | 28.7 | 27.2 |
|  | 40 | 86.3 | 67.4 | 48.6 | 36.7 |
|  | 60 | 97.5 | 78.5 | 72.4 | 43.9 |
|  | 80 | 98.1 | 80.4 | 76.6 | 52.1 |
|  | 100 | 98.1 | 82.3 | 79.2 | 48.6 |
| Standby power (kW) | 3 | 4.0 | 7.2 | 11.8 | 14.9 |
|  | 6 | 3.8 | 5.0 | 12.5 | 13.8 |
|  | 9 | 3.9 | 7.1 | 12.3 | 12.7 |
|  | 12 | 4.2 | 7.5 | 12.4 | 14.1 |
|  | 15 | 4.3 | 7.6 | 13.0 | 14.8 |
|  | 18 | 4.1 | 7.4 | 13.2 | 15.0 |
|  | 21 | 3.9 | 7.2 | 12.9 | 14.7 |
|  | 24 | 3.8 | 7.0 | 12.6 | 14.5 |
